# Attentional Biases Towards Body-Related Stimuli in Healthy Males: A Systematic Review

**DOI:** 10.1177/00332941231177243

**Published:** 2023-05-21

**Authors:** Alexandra S. Kirby, Rebecca Jenks, Francesca Walsh, Michael Duncan

**Affiliations:** 1 Centre for Sport, Exercise and Life Sciences, 2706 Coventry University, Coventry, UK; 2 Department of Arts, Humanities and Human Sciences, 1713 Newman University, Bartley Green, UK; 3 School of Psychological, Social and Behavioural Sciences, 2706 Coventry University, Coventry, UK

**Keywords:** body image, attentional bias, males, systematic review, body-related stimuli

## Abstract

Recent literature has discussed the role of attentional biases towards body-related stimuli. Specific foci have been on those with high levels of body image concerns and female samples. Unfortunately, there has been limited focus on male samples within existing literature. The aim of the current study was to provide a critical synthesis of the findings of existing studies exploring attentional biases in adult males towards body-related stimuli. Critical synthesis of the findings of 20 studies explored four key methodologies: eye-tracking, dot-probe, visual search, and other methodologies (e.g. ARDPEI task). The current review provides evidence of specific attentional biases towards body-related stimuli in adult males experiencing body image concerns. Similar patterns of attentional biases are also demonstrated in males with body image pathologies. However, there appears to be distinct patterns of attentional biases for male and female participants. It is recommended that future research considers these findings and utilises measures developed specifically for male samples. Furthermore, additional variables require further attention, i.e. reasons for engaging in social comparison and/or engaging in physical activity.

Body dissatisfaction is a predictor of the onset of eating disorders, in addition to other psychopathologies and unhealthy behaviours, e.g. depression, anxiety and unhealthy weight-control behaviours (Loughnan et al., 2015; Porras-Garcia et al., 2020a; Rodgers & DuBois, 2016). In recent years, interest has grown regarding the role that cognitive biases play within the formation and maintenance of body dissatisfaction, and how these biases can inform the development of effective interventions (Glashouwer et al., 2016; Jiang & Vartanian, 2018; Kerr-Gaffney et al., 2019; Rodgers & DuBois, 2016; Uusberg et al., 2018). It is widely agreed within current literature that attentional biases play a key role in body dissatisfaction (e.g., Jiang & Vartanian, 2018; Lane et al., 2017; Rodgers & DuBois, 2016). Results of prior studies demonstrate that individuals with body dissatisfaction selectively attend to schema-relevant (e.g. appearance-related) information over more neutral information, which results in, and maintains, negative emotions and self-image (Jiang & Vartanian, 2018; Kerr-Gaffney et al., 2019; Lane et al., 2017; Loughnan et al., 2015; Lyu et al., 2019; Porras-Garcia et al., 2019; Rodgers & DuBois, 2016).

Previous research on this topic has primarily focussed on attentional biases within female participants, in both eating disorder sample groups (Kerr-Gaffney et al., 2019; Naumann et al., 2019; Uusberg et al., 2018) and/or healthy controls (Allen, J. L., Mason, Stout, & Rokke, 2018a; Bauer et al., 2017; Glashouwer et al., 2016). In a recent review of eye-tracking research, Kerr-Gaffney et al. (2019) discussed attentional biases towards both food- and body-related stimuli with an almost exclusively female population. Several studies within the review provided evidence for increased attention to food-related images in individuals with eating disorders compared to healthy controls. Furthermore, findings supported the proposal that negative body image schemas relating to body dissatisfaction cause cognitive biases, which lead to negative emotions and reinforce negative body image schemas. Several studies in Kerr-Gaffney et al.’s (2019) review demonstrated that the more body-dissatisfied participants were, the stronger their attentional biases were towards both food- and body-related stimuli; a consistent finding across both eating disorder and healthy control groups. However, while it is clear that attentional biases towards schema-relevant information in body image exist, there is great variability in the methodologies employed and the attentional processes measured (Jiang & Vartanian, 2018; Kerr-Gaffney et al., 2019; Rodgers & DuBois, 2016). Furthermore, the resounding conclusion within this field is that additional research with a male sample is needed as this is currently lacking (Allen, J. L. et al., 2018a; Allen, L. et al., 2018b; Rodgers & DuBois, 2016; Uusberg et al., 2018). This is particularly important as recent research points towards higher levels of body dissatisfaction among men to the point that this is now considered normative (e.g., Boyd & Murnen, 2017; Hargreaves & Tiggemann, 2009; Jankowski et al., 2018).

Over the last decade, body image research has increasingly focused its attention to exploring male body image and the impact that viewing the ‘ideal male body’ in the media is having on males of all ages (e.g., Gattario et al., 2015; Grogan & Richards, 2002; Hargreaves & Tiggemann, 2009; McNeill & Firman, 2014). The ‘ideal male body’ portrayed in media has become more muscular over time, with the current ideal depicting a man with a mesomorphic, V-shaped body type: muscular chest and arms with a slim, lean waist (Boyd & Murnen, 2017; Murray et al., 2017). In a review by Murray et al. (2017) it was found that this body ideal has been displayed by various ‘male role models’ in different media, including screen stars, models, action figures and computer game characters. As a result of this depiction a preference for more muscular body types is being found amongst males (Baghurst et al., 2007; Grogan & Richards, 2002; Hargreaves & Tiggemann, 2009). Unfortunately, this preference towards the muscular ideal is associated with several risk factors, including the development of muscle dysmorphia (MD; Olivardia et al., 2000; Pope et al., 1997), anabolic-androgenic steroid use (Olivardia et al., 2000; Pope et al., 1997, 2012), and excessive exercise (Dawson & Hammer, 2020; Tod & Edwards, 2015). While such risk factors may be present for females, they are more predominantly noted amongst males as they typically demonstrate a greater drive for muscularity (Dawson & Hammer, 2020; Olivardia et al., 2000; Tod & Edwards, 2015).

The preference for, paired with the increasing prevalence of, the muscular ideal may lead to biased attention towards such stimuli, particularly in body-dissatisfied individuals. Rodgers and Dubois (2016) posit that, when stimuli are “perceived as a reflection of the environment” (e.g., idealised other bodies portrayed within the media), upward social comparison processes are initiated and exacerbate body dissatisfaction (p. 9). Recent research has further demonstrated this in both males and females through serial mediation models (Dondzilo, Mills, & Rodgers, 2021b; Dondzilo et al., 2021a). Within these serial mediation models, the relationship between increased attentional engagement and higher levels of body dissatisfaction is mediated by upward social comparisons prompted by engagement with idealised body images (both peer and media images). These social comparisons were also associated with rumination that the individual does not meet the ideal appearance standards and higher levels of body dissatisfaction (Dondzilo et al., 2021a, 2021b).

While sociocultural models of body dissatisfaction are widely used to explain media effects of body image (e.g., Bailey & Ricciardelli, 2010; Blechert et al., 2009; Rodgers & DuBois, 2016), alternative explanations may come from cognitive theories. From a cognitive-behavioural perspective, it is proposed that those experiencing body dissatisfaction experience maladaptive appearance-related schemas which result in cognitive biases (Altabe & Thompson, 1996; Higgins, 1987; Markus et al., 1987; Rodgers & DuBois, 2016). Such biases include selective attention, memory biases, and judgement biases towards schema-relevant stimuli (e.g., images depicting media ideals). Williamson et al. (1999; 2004) developed the cognitive information-processing model of body image. This model posits that cognitive biases are a function of disordered body schema, not disordered eating behaviour, and therefore can develop in healthy individuals who display a preoccupation with body size and shape. When activated, these cognitive biases guide an individual’s cognitive processing, resulting in interpretations which support the original cognitive schema and therefore perpetuating the cognitive bias.

Alternatively, media effects of body image may be explored through objectification theory (Daniel & Bridges, 2010; Fredrickson & Roberts, 1997). While objectification theory was initially developed to explain female body image concerns (Fredrickson & Roberts, 1997), more recent research suggests this theory could be used to explain male body image concerns (e.g., Aubrey, 2006; Daniel & Bridges, 2010; Heath et al., 2016). Although men may not typically experience sexual objectification directly from the gaze of women, the increase of the sexual objectification of men in the media has been associated with increased self-objectification and body surveillance (Aubrey, 2006; Daniel & Bridges, 2010; Heath et al., 2016). Specifically, Heath et al. (2016) hypothesise that the body image variables utilised within the self-objectification model may differ between genders, with muscle dysmorphia characteristics playing a larger role than factors such as body shame. The authors suggest an alternative hypothesis that these differences are not down to gender differences, but instead differences in the ideal aesthetic (Heath et al., 2016). Further research is required to explore this hypothesis.

Different methodologies have been used to measure cognitive and attentional biases in body image research, with recent reviews discussing the frequency, findings, and strengths and limitations of each paradigm (Jiang & Vartanian, 2018; Rodgers & DuBois, 2016). One of the most widely used paradigms is eye-tracking, where participants gaze is measured while viewing appearance related stimuli (Rodgers & DuBois, 2016). Findings from eye-tracking studies have typically observed similar gaze patterns between clinical and healthy control samples, with participants who have a more negative perception of their own body demonstrating greater attention towards unattractive areas of their own bodies (Jiang & Vartanian, 2018). However, findings using other body stimuli or aiming to confirm results of studies that have used alternative paradigms have been mixed. Another widely used paradigm is the dot-probe task, where participants reaction times towards a probe that replaces body-related or neutral stimuli (Rodgers & DuBois, 2016). Findings from dot-probe studies have observed inconsistencies in findings across studies, particularly between clinical and non-clinical samples (Jiang & Vartanian, 2018). Many of the studies reviewed have utilised female participants, so it is unclear from these existing reviews what patterns of attentional bias exist within a male sample.

Considering that male body dissatisfaction can now be regarded as normative, and that there appears to be a direct link between body dissatisfaction and biased attentional processes in females, it is important that research looks to understand the specific attentional biases experienced by body dissatisfied males. To date, only one review (Talbot & Saleme, 2022) has explored attentional biases towards body-related stimuli within males. However, Talbot and Saleme’s (2022) review focusses solely on males’ attentional biases towards body-related stimuli demonstrating the muscular ideal and biases towards the self. The current systematic review aims to provide a critical synthesis of the findings of existing studies exploring attentional biases in adult males towards body-related stimuli, including other areas of body image concern such as height, and will explore differences and similarities between males/females and healthy males/males with body image pathologies. A review of this nature is needed to provide a robust foundation so evidence-based interventions can be targeted to this population. Through this synthesis, the current review aims to answer the following questions:

• Are attentional biases towards body-related stimuli evident in healthy adult males with high-levels of body-image concern?
• What specific attentional patterns are evident in healthy adult males with high-levels of body-image concern when viewing body-related stimuli?
• How does this compare to other groups of interest such as healthy adult males with no/low-levels of body-image concern or adult males with body image pathologies (e.g., muscle dysmorphia)?
• How do these findings compare with female participant groups? Do any studies provide a direct comparison?
• How have attentional biases in adult males been studied? What methodologies have been employed? (e.g., measures of body (dis)satisfaction, stimulus type, attentional bias paradigm)

## Method

A systematic review of the literature focussing on attentional biases towards body- and food-related stimuli in males was conducted following the PRISMA guidelines (Page et al., 2021). A review protocol is available at https://www.crd.york.ac.uk/prospero/(Registration Number: CRD42021233662). Ethics approval was obtained through Coventry University’s Ethics Team (Appendix A).

### Eligibility Criteria

Literature was eligible for inclusion in the current systematic review if it focussed on attentional biases towards body-related stimuli in adult males. Literature was excluded if participants were adolescents or children (under 18 years of age) or older adults (over 70). While the focus of the current systematic review is on healthy adult males with no diagnosed eating/body image disorders, literature utilising adult male participants with diagnosed eating/body image disorders was included as a comparison group. Literature including adult female participants (with or without a diagnosed eating/body image disorder) was also included in the review, but only if male participants were also present in the same study and gender differences assessed to determine specific gender biases. If males contributed <25% of the sample, studies were excluded. No limitations were placed on the attentional bias paradigms/methodologies included in the current review, nor on the total sample size of the study.

Due to the paucity of literature in this area of research, no date limitations were applied to the literature searches. Both published and unpublished literature was considered for review. Literature must be written in English to be included in the current review.

### Information Sources and Search Strategy

Literature searches were carried out on the following databases: EBSCOhost databases (Academic Search Complete, CINAHL Complete, APA PsycARTICLES, MEDLINE, APA PsycINFO) were searched on Sunday 6th November 2022, SCOPUS was searched on Monday 7th November 2022, and Grey Literature Databases (EThOS, ProQuest Dissertation and Theses, World Cat) were searched on Monday 7th November 2022.

The following search terms were used: (“body image” OR “body perception” OR “body *satisfaction” OR “body image disturbance” OR “body dysmorphia” OR “body dysmorphic disorder” OR “self-perception” OR “body esteem” OR “body awareness” OR “body mass index” OR “personal appearance” OR “body size” OR “eating behaviour*” OR “eating disorder”) AND (“attention* bias” OR “cognitive bias*” OR “dot probe” OR “visual search” OR “visual tracking” OR “electroencephalography” OR “EEG” OR “eye tracking” OR “eye tracking technology”) AND (“adult*” OR “human male*” OR “psychology of men” OR “masculinity” OR “male attitude*” OR “male”).

Results were additionally filtered for language (English). Literature was collected with matching search terms in the title, abstract or key words. The final search was conducted on Monday 7th November, 2022 by the principal investigator (PI). Search results were then cross-checked and confirmed by team members.

### Study Selection

Literature from the searches were combined and duplicates were removed by hand by the PI. Article titles and keywords were screened initially, and relevant literature was recorded. Abstracts of these recorded articles were then reviewed by the PI and MD to determine which articles would be eligible for a full-text review. Full-text articles were reviewed by the PI, and reference sections of these articles were hand-searched for any additional, relevant studies.

### Data Collection Processes

The PI initially applied the eligibility criteria and selected studies for inclusion in the current systematic review. Details of studies were logged in Microsoft Excel alongside decisions regarding inclusion and reasons for exclusion (if applicable). The PI then extracted data from the included studies and recorded it in an Excel spreadsheet. These data included: Authors & publication date; participant details; paradigm/methodology; stimuli type; grouping variable(s); dependent variable(s); findings (see Table 1 in the Results section). Data were then checked by team members (RJ and FW) to ensure accuracy and provide consensus on eligibility decisions.

**Table 1.** Characteristics of Included Studies.

Authors & Publication Date	Participant Details	Paradigm/Methodology	Stimuli Type	Grouping Variable(s)	Dependent Variable(s)	Findings
Bernard et al. (2018)	65 USA university students (55% male), mean age = 19.82, 85% Caucasian	Eye-tracking	High versus medium versus low ideal male body shape images		Dwell time and first fixation time	Appearance-focused participants fixated on arms and stomachs for longer and more quickly and faces for less time than personality-focused participants. Participants fixated on faces of average body shapes more than high and low ideal body shapes, on arms of high ideal more than average and low ideal, and on stomachs of low ideal for longer and more quickly than average and high ideal. Effects not moderated by gender.
Cai et al. (2020)	44 Chinese adult males, mean age = 20.14	Electroencephalogram (EEG), dot-probe	tall-related versus short-related versus neutral words (audio stimuli); Gabor patches (visual stimuli)	NPS-S > 2 versus NPS-S < 1	Reaction time, accuracy, ERP amplitudes	Significant interaction for accuracy for word type x group, with HHD participants showing greater accuracy for visual targets preceded by tall-related words than LHD participants. Short-related words elicited larger N1 amplitudes than tall-related and neutral words. Tall-related words elicited more negative N2ac amplitudes than short-related words. Significant interaction for N2ac amplitude for word type x group, with tall-related words eliciting significantly larger amplitudes than short-related and neutral words within the HHD group, but not the LHD group. Tall-related words also elicited significantly larger N2ac amplitudes for the HHD group than the LHD group.
Chen et al. (2017)	47 male undergraduate and graduate students at Southwest University in China, mean age = 21.34 years	Dot-probe, eye-tracking	tall-related versus short-related versus neutral words	NPS-S > 2 versus NPS-S < 1	Reaction time, direction bias, first fixation latency bias, first fixation duration bias, gaze duration bias	HHD group demonstrated stronger attention avoidance to short-related words than LHD group and an initial avoidance bias from height-related words. Compared to LHD group, HHD group had a shorter first fixation duration towards short-related words and were more likely to disengage from height-related words.
Cho and Lee (2013)	88 Korean undergraduates (51% male), mean age = 21.80 years	Eye-tracking	thin versus normal versus muscular versus fat bodies (male and female)	For males: EDI-2 < 2.56 versus EDI-2 > 4.00 For females: EDI-2 < 3.00 versus EDI-2 > 4.67	Gaze durations, fixation frequency	High BD males demonstrated longer gaze duration for muscular bodies than other body types compared to low BD males. High BD females demonstrated longer gaze duration for thin bodies compared to low BD females, but not for other body types. High BD males demonstrated higher fixation frequencies for muscular bodies than normal bodies compared to low BD males. High BD females demonstrated higher fixation frequencies for thin bodies than normal or fat bodies compared to low BD females.
Cordes et al. (2016)	45 German males, mean age = 28.7 years	Eye-tracking	own versus normal versus muscular versus hyper-muscular male body images	Drive for thinness, drive for muscularity	Gaze behaviour	The chest and abdominal regions received the most visual attention (50–59%) across all body stimuli. High-DT men demonstrated longer gaze on self-reported unattractive areas of their own body and shorter gaze on self-reported attractive areas then low-DT men. High-DM men demonstrated greater attentional biases towards self-reported attractive areas of their own body than low DM men. All men demonstrated greater attentional biases towards attractive areas of the muscular bodies than unattractive areas.
Cordes et al. (2017)	49 weight-training German male students, mean age = 28.7	Eye-tracking	own versus normal versus muscular versus hyper-muscular male body images		Attentional bias, state body image, negative affect	Exposure to images of both own and muscular body images led to a decrease in state body images, with own body images also leading to an increase in negative affect scores. Pictures of normal and hyper-muscular bodies also had a negative effect on state body images, but to a lesser degree than own and muscular images. Males who demonstrated an attentional bias to their own self-reported negative body areas demonstrated more negative affect and state body image after viewing images than those with a more positive/neutral viewing pattern.
Dondzilo et al. (2021b)	66 Australian male undergraduates, mean age = 20.65	ARDPEI Task	muscular male bodies versus non-muscular male bodies versus abstract art images		Engagement bias index, disengagement bias index	Support for serial mediation model (engagement bias to muscular bodies - > appearance comparisons - > ED-specific rumination - > body dissatisfaction)
Hewig et al. (2008)	51 German university students (51% males), mean age = 21.5	Eye-tracking	Male and female images: fully clothes versus undershirts versus underwear and swimwear versus topless versus nudes versus backside views	Participant gender	EDI-2 scores, total contact time, number of fixations	Males had significantly lower drive for thinness scores than females. Participants with higher drive for thinness looked more often and for longer at waist, hips, legs, and arms and less often/shorter to head/face. This effect strongest for undershirts category and underwear/swimwear, and weakest for nudes. No moderating gender effects. Males demonstrated more varied and stronger results than females who generally avoided looking at face/head. Males scoring higher on drive for thinness showed increased total contact time, this was not observed in females higher on drive for thinness.
Jin et al. (2018)	65 weight-training Chinese adult males, mean age = 23.66	Eye-tracking, visual probe task	Bodybuilder (large musculature) versus bodybuilder (small musculature) versus car exterior (neutral) images	HRMD (CMASS score 51-72), LRMD (CMASS score 24-42)	Reaction time, gaze direction, first saccade latency, first fixation duration	Both HRMD and LRMD groups demonstrated attentional bias towards large muscle images, with HRMD group attending to these images and LRMD group avoiding these images. All participants demonstrated gaze direction bias towards large muscle images. HRMD group also showed gaze direction bias for small muscle images. LRMD group demonstrated slower attention orientation towards body images (small or large muscle) than HRMD group. HRMD group demonstrated maintained attention towards large muscle images, while LRMD group demonstrated initial avoidance towards these images. HRMD group also maintained attention for longer for large muscle images compared to small muscle images.
Lane et al. (2019)	60 Australian adult males, mean age = 31.98	Dot-probe task	Image cues: attractive male body versus neutral forest images Word stimuli: negative-appearance, versus positive-appearance versus neutral words	DPT condition	Attentional bias	No effect of DPT condition or word type on attentional bias scores. Attentional bias towards positive-appearance words positively associated with perceived pressure from media, internalisation of leanness ideal and leanness dissatisfaction for appearance-cued DPT.
Liu et al. (2014); Study 1	68 male Chinese students, mean age = 21.25	Dot-probe task	Tall stature versus short stature versus neutral words	NPS-S	Reaction time	HSD group had significantly slower RTs for probes following short-stature words compared to LSD group. No difference between groups for RTs following tall-stature words.
Liu et al. (2014); Study 2	67 male Chinese students, mean age = 22.81	Attention shifting task, recognition task	Tall stature versus short stature versus neutral words	NPS-S	Reaction time, percent correct, memory sensitivity, response bias	Overall sample slower to judge short stature words than tall stature and neutral words. HSD group demonstrated greater recognition accuracy and higher recognition sensitivity for short stature words than LSD group. No response biases towards stature related words found.
Nikkelen et al. (2012)	50 male Dutch undergraduate students, mean age = 23.1)	Eye-tracking	normal versus muscular versus body builder body images		Mean dwell time, total dwell time, total number of fixations, total number of first fixations	Mean body dissatisfaction did not differ between participants in either media exposure group. Moderating effect of visual attention to the abdomen, with high visual attention to the abdomen being related to high body dissatisfaction in the neutral condition, and low visual attention to the abdomen being related to high body dissatisfaction in the experimental condition.
Pazhoohi et al. (2019)	82 Portuguese undergraduate students (39% male), mean age = 20.90	Eye-tracking	Male and female 3D models: low versus intermediate versus high SHR variations	Participant gender	Dwell time, fixations, attractiveness ratings	Males rated male images with higher SHRs as more attractive than lower SHRs for both front and back views, and intermediate SHR as more attractive for front view female images. Females rated intermediate SHRs as more attractive for both male and female images. Males demonstrated longer dwell times on the chests of male images with higher SHRs than lower SHRs, but not for female images. Females demonstrated no differences in dwell time on the chests of male or female images in any SHR group.
Porras-Garcia et al. (2019)	85 Spanish university students (47% male), mean age = 24.7	Eye-tracking, virtual reality	Virtual bodies, real size and larger size (40% larger than participant)	High versus low body dissatisfaction, participant gender	Number of fixations, complete fixation time	Women demonstrated higher number of fixations and looked for longer at the weight related AOIs than males. Males demonstrated an attentional bias towards non-weight related AOIs. Men demonstrated longer gaze towards muscular-related AOIs, while women demonstrated a preference for non-muscular AOIs.
Porras-Garcia et al. (2020b)	40 male Spanish university students, mean age = 24.35	Eye-tracking, virtual reality	Virtual avatars of participants	High versus low muscularity dissatisfaction	Number of fixations, complete fixation time	Males with high levels of MD looked at muscle-related body areas for longer and with greater frequency than those with low levels of MD. Specific interest was shown towards the shoulders and chest.
Talbot et al. (2019)	63 Australian male undergraduate students, mean age = 21.91	Compound visual search task	Average versus obese versus muscular body stimuli		Reaction time, accuracy	Reaction times for the muscular-congruent and obese-congruent trials significantly faster than neutral trials. Muscular-congruent trials had significantly faster reaction times than obese-congruent trials. Muscular attentional bias scores positively correlated with muscularity dissatisfaction, dietary restraint, and shape concern. Obese attentional bias scores positively correlated with dietary restraint. Obese-incongruent reaction times positively correlated with body fat dissatisfaction, shape concern, weight concern.
Unterhalter et al. (2007)	40 UK undergraduate students (50% male), mean age = 21.3 years	Memory bias task	positive weight/shape versus negative weight/shape versus positive muscle versus negative muscle versus neutral body versus non-body related words		Number of words recalled	Men recalled significantly more muscle words than women, while women recalled significantly more weight words than men. Both sexes demonstrated better recall for positive words, but women recalled significantly more negative words than men.
Waldorf et al. (2019)	72 adult males, mean age = 23.9 years	Eye-tracking	own versus average versus lean-muscular versus hyper-muscular body images	MD diagnosis versus weight training control versus non-weight-training control	Total dwell time	MD group and non-weight training controls demonstrated significantly longer dwell times on subjectively unattractive areas of own body. All participant groups gazed longer at the unattractive areas of average body. MD group and non-weight training controls demonstrated significantly longer dwell times on subjectively attractive areas of lean-muscular body. MD group demonstrated significantly longer dwell times on attractive areas of hyper-muscular body.
Warschburger et al. (2015)	58 German adults (41% male), mean age = 26.2	Eye-tracking	Own versus BMI-matched control versus gender-matched control images	Participant gender, participant BMI (underweight: BMI < 18.5 kg/m^2^, lower normal weight: BMI 18.5–20.4 kg/m^2^, medium normal weight: BMI 20.5–22.4 kg/m^2^, higher normal weight: BMI 22.5–24.9 kg/m^2^, overweight: BMI 25.0–29.9 kg/m^2^, obese: BMI ≥ 30 kg/m^2^)	Percentage of fixations for attractive, neutral, and unattractive regions of interest	Women demonstrated longer gaze on attractive regions of interest than men, while men looked significantly longer an unattractive regions of interest. Analyses of attentional bias did not detect significant differences between males and females. Overweight participants demonstrated a longer gaze towards attractive regions of interest and shorter gaze at unattractive regions of interest than normal weight participants.

Note: NPS-S: Negative Physical Self Scale-Stature Concerns subscale (Chen, H. et al., 2006); HHD: high levels of height dissatisfaction, LHD: low levels of height dissatisfaction (Cai et al., 2020; Chen, F. et al., 2017); EDI-2: Eating Disorder Inventory-2 (Garner, D. M., 1991); BD: body dissatisfaction (Cho & Lee, 2013); DT: drive for thinness, DM: drive for muscularity (Cordes et al., 2016); ARDPEI task: Attentional Response to Distal versus Proximal Emotional Information task (Grafton & MacLeod, 2014); ED-specific: eating-disorder specific (Dondzilo et al., 2021a); CMASS: Chinese version of the Muscle Appearance Satisfaction Scale (Jin et al., 2015); HRMD: high risk of muscle dysmorphia, LRMD: low risk of muscle dysmorphia (Jin et al., 2018); DPT: dot-probe task (Lane et al., 2019); HSD: highly stature dissatisfied, LSD: less stature dissatisfied (Liu et al., 2014); SHR: shoulder to hip ratio (Pazhoohi et al., 2019), AOI: area of interest (Porras-Garcia et al., 2019); MD: muscularity dissatisfaction (Porras-Garcia et al., 2020b); MD: muscle dysmorphia (Waldorf et al., 2019).

### Synthesis Methods

Data were synthesised using a qualitative approach, whereby findings will be discussed in relation to specific attentional bias methodologies used within the selected studies. There were no minimum number of studies required for data to be synthesised under a specific methodology heading.

## Results

### Study Selection

**Figure 1.**
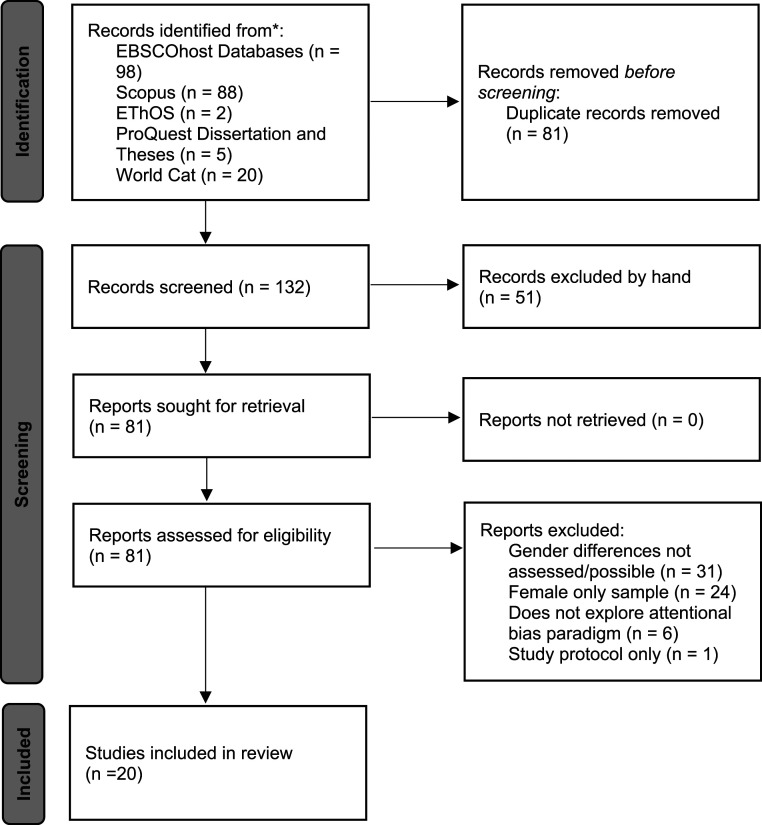
Systematic review flow diagram illustrating the number of articles at each given stage in the review process.

A total of 213 articles were retrieved following database searches, with 81 duplicate records being removed. The titles and abstracts of the remaining 132 articles were reviewed, with 51 studies being excluded at this stage as they did not meet the eligibility criteria. The remaining 81 articles were assessed for eligibility. Articles were excluded from the current review for the following reasons: a) gender differences were not assessed within the paper or not possible due to small percentage of male participants, b) studies included a female only sample, c) the articles did not utilise an attentional bias paradigm, or d) the article contained a study protocol only. Following the final eligibility screening, 20 articles were found to meet the eligibility criteria of the current study and were included in the final critical synthesis (Figure 1, Table 1).

### Risk of Bias

**Figure 2.**
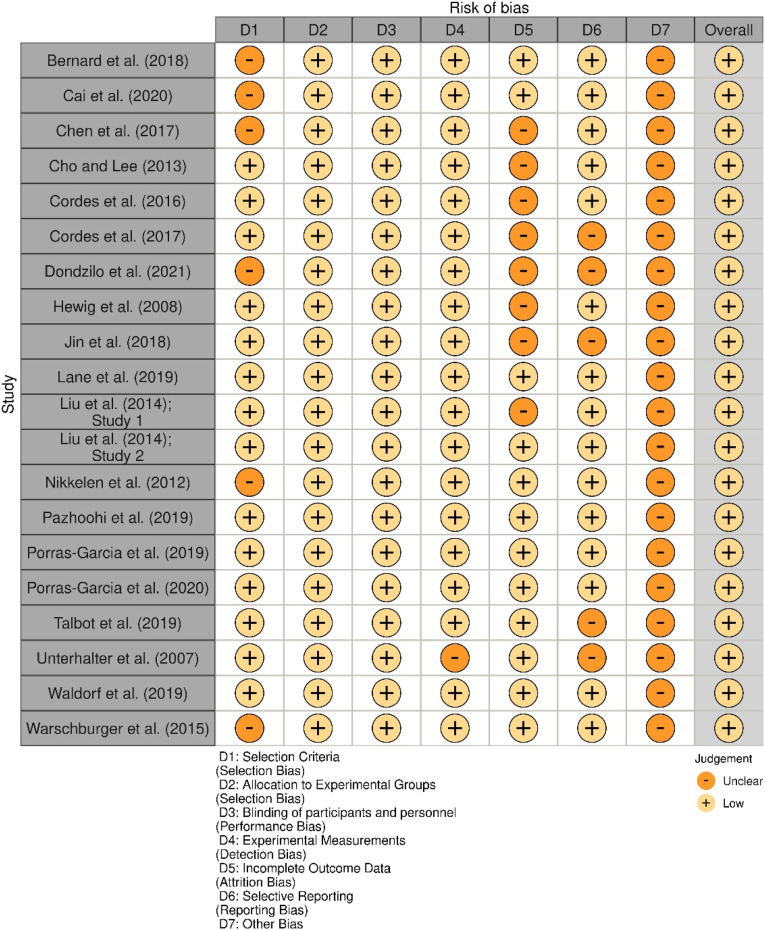
Robvis Visualization Tool (McGuinness & Higgins, 2020) output showing Risk of Bias Assessment.

Risk of bias was assessed by the PI, with team members (XX and XX) providing additional judgements where there were any concerns regarding the reliability of the data selected. The main characteristics of interest for each study included in the current review were the methodologies utilised by the research team, the participants included in each study and the overall findings of the study. Risk of bias assessments were carried out at a study level as the current review aims to explore the different outcomes that currently exist, rather than support a specific outcome/intervention. Risk of bias was completed using the Generic Template of the *robvis* Visualization Tool (McGuinness & Higgins, 2020). Bias was assessed for the following domains: selection criteria, allocation to experimental groups, blinding of participants and personnel, experimental measurements, incomplete outcome data, selective reporting, and other potential biases (see Figure 2 below).

### Findings From Eye-Tracking Studies

Most studies (n = 12) included in the current review used an eye-tracking methodology. These studies presented participants with visual body stimuli and measured number of fixations, dwell times, and general gaze patterns. Overall, males generally demonstrated longer gaze durations when viewing muscular images (Cho & Lee, 2013; Jin et al., 2018; Porras-Garcia et al., 2019) compared to other body stimuli. Specifically, participants demonstrated longer gaze durations for arms (Bernard et al., 2018; Porras-Garcia et al., 2019), chest (Cordes et al., 2016; Porras-Garcia et al., 2019, 2020b), shoulders (Porras-Garcia et al., 2019, 2020b), and/or abdomen (Cordes et al., 2016; Porras-Garcia et al., 2019) regions of body stimuli.

Considering attention towards muscular bodies, multiple studies have built upon findings that the muscular ideal only affects a select subset of males rather than the entire population (Jin et al., 2018; Lane et al., 2019; Nikkelen et al., 2012; Porras-Garcia et al., 2020). For example, weight-training Chinese males at high risk of muscle dysmorphia displayed attentional biases towards muscular images, particularly high musculature images, responding more quickly and looking at the images for longer than those at low risk of muscle dysmorphia (Jin et al., 2018). Furthermore, Spanish males with high muscle dissatisfaction demonstrated greater attentional biases towards muscle-related body areas of a virtual avatar, specifically the chest and shoulders, while those with low muscle dissatisfaction demonstrated no significant attentional biases towards muscle-related body areas (Porras-Garcia et al., 2020b). In both studies, it is suggested that males who are low risk of muscle dysmorphia/have low muscle dissatisfaction scanned over the whole body of the stimuli they were presented with (Jin et al., 2018; Porras-Garcia et al., 2020b). However, while males with low muscle dissatisfaction did not demonstrate significant attentional biases towards muscle-related body areas, all males regardless of muscle dissatisfaction demonstrated higher levels of attention towards the chest and abdomen of virtual avatars than other areas of interest (Cordes et al., 2016; Porras-Garcia et al., 2020b). It is possible that these areas relate to other dimensions of body image dissatisfaction, such as body fat or masculinity concerns, and that future studies should explore a range of dimensions beyond muscularity satisfaction.

In a sample of German males with either high/low drive-for-thinness or high/low drive-for-muscularity, Cordes et al. (2016) found differing body-directed gaze behaviours depending on the type and level of body dissatisfaction experienced by participants. Males with high drive-for-muscularity demonstrated greater attentional biases towards self-reported attractive areas of their own body than males with low drive-for-muscularity. In contrast, males with high drive-for-thinness demonstrated longer gaze towards self-reported unattractive areas of their own body and shorter gaze towards self-reported attractive areas than males with low drive-for-thinness. Despite this distinct pattern, all groups demonstrated greater attention to attractive areas of the muscular body stimuli than unattractive areas; suggesting that, while muscularity is not the only dimension of male body image, it is regarded by males as a key element of male body image and the body image ideal (Cordes et al., 2016).

Building upon their previous findings, Cordes et al. (2017) carried out an eye-tracking study with weight-training, German male students. They found that exposure to muscular images led to decreased state body satisfaction, with images of one’s own body leading to the greatest effects of decreased state body satisfaction and increased negative affect. It is suggested that images of the self are more likely to activate cognitive biases, which in turn elicit negative affect (Williamson et al., 2004). Unfortunately, Cordes et al. (2017) did not examine the differences between weight-training and non-exercising males, nor did they establish participants motivation for exercise (i.e., to improve appearance, athletic ability, health). These factors are important for understanding the underlying mechanisms of body dissatisfaction when exposed to body images. For example, weight-training males may place greater value on muscularity than non-weight training males, leading to stronger attentional biases towards muscular images.

Further eye-tracking studies found attentional biases to be a moderator of the effect of exposure to media images on body dissatisfaction. Sociocultural models of body image suggest that media influences play a key role in shaping body image concerns (Murray et al., 2017). There is considerable evidence that those dissatisfied with their appearance engage in higher levels of appearance-based social comparison (e.g., Myers & Crowther, 2009; Uusberg et al., 2018). As this social comparison is typically an upwards comparison (e.g., Bailey & Ricciardelli, 2010), and evidence suggests body dissatisfied individuals tend to demonstrate more upward comparisons when viewing other’s bodies and idealised media images (e.g., Hewig et al., 2008; Jansen et al., 2005), the authors propose that attentional biases play an important role in the relationship between idealised media images and body dissatisfaction.

In a 2012 eye-tracking study, Nikkelen et al. found that mean body dissatisfaction did not differ between participants after viewing either a neutral or muscular-ideal commercial. However, there was a moderating effect of visual attention to the abdomen; with high visual attention being related to high body dissatisfaction in the neutral condition, and low visual attention being related to high body dissatisfaction in the experimental condition. Nikkelen et al. (2012) suggest these findings may be indicative of differing motives for engaging in social comparison; self-evaluation or self-improvement. According to social comparison theory, individuals are driven towards upward comparisons in relation to abilities and attributes which can be improved (Festinger, 1954). When engaging in social comparison from a self-evaluation perspective, an individual will evaluate their own features against those of another (e.g. a media ideal) which may lead to feelings of dissatisfaction with themselves. Alternatively, when engaging in social comparison from a self-improvement perspective, an individual will focus on how they can improve themselves in order to achieve the standard of another (e.g. a media ideal) which may lead to feelings of inspiration rather than dissatisfaction (Nikkelen et al., 2012). Nikkelen et al. (2012) speculate that the relationship between low visual attention and body dissatisfaction in the experimental condition was a result of participants adopting a self-improvement motive of social comparison (p.318). Males who demonstrated higher levels of attention to the abdomen were possibly preoccupied with changing their body to boost their masculinity, thus focussed on how to improve their bodies to achieve the media ideal.

Furthermore, studies exploring attentional biases in females when viewing own and others’ bodies have demonstrated participants tend to focus on their own unattractive and others’ attractive body areas (Blechert et al., 2009; Jansen et al., 2005; Roefs et al., 2008). Similar patterns have been found in studies with male participants. In a study of adult males with an MD diagnosis, healthy weight-training controls, or healthy non-weight training controls, Waldorf et al. (2019) utilised eye-tracking technology to explore total dwell time when viewing their own, average, lean-muscular and hyper-muscular images. While all participants demonstrated longer gaze patterns at the subjectively unattractive areas of the average body, the MD and non-weight training control groups also demonstrated significantly longer dwell times on the subjectively unattractive areas of their own bodies and the subjectively attractive areas of the lean-muscular body. Furthermore, the MD group also demonstrated significantly longer dwell times on subjectively attractive areas of the hyper-muscular body. These gaze patterns were not observed in the weight-training control group, suggesting body image pathology may play a greater role in negative body-image biases than drive for muscularity. Alternatively, it may be that weight-training controls focus more on subjectively positive body areas as a measure of training success (Cordes et al., 2016; Waldorf et al., 2019). Interestingly, all participant groups were negatively affected by images of their own body (Waldorf et al., 2019). However, only the MD group demonstrated a significant negative effect of viewing the hyper-muscular body image. These findings highlight the importance of the subjective “ideal male body type”, particularly when working with participants with body image pathologies.

The remaining eye-tracking studies explored differences in male and female gaze patterns when viewing body-related stimuli. Multiple eye-tracking studies (n = 4) reviewed demonstrated support for gender differences. For example, a study conducted with male and female Korean undergraduate students found that males with high levels of body dissatisfaction demonstrated a longer gaze duration and higher fixation frequencies for muscular bodies than other body types (Cho & Lee, 2013). On the other hand, females with high levels of body dissatisfaction demonstrated longer gaze durations and higher fixation frequencies for thin bodies. Furthermore, in an eye-tracking study with Spanish university students, male participants demonstrated greater attentional biases towards non-weight and muscle-related areas of interest, while female participants demonstrated greater attentional biases towards weight-related and non-muscular areas of interest (Porras-Garcia et al., 2019). These findings suggest different ideals for male and female body image, with males showing preference for a muscular ideal rather than a thinner/weight-related ideal – possibly because males tend to focus on functionality rather than aesthetics (Lane et al., 2019).

While it is clear that males and females identify with different media ideals (e.g., Cho & Lee, 2013; Porras-Garcia et al., 2019), it could be argued that these findings do not demonstrate different attentional patterns between males and females. Instead, these findings could be interpreted as both males and females demonstrating the same attentional patterns to media ideals. However, additional eye-tracking studies have found that males and females display different viewing patterns (Hewig et al., 2008; Pazhoohi et al., 2019). For example, Hewig et al. (2008) found that, while all participants high in drive for thinness demonstrated longer and more frequent viewing of waist, hips, legs and arms, males generally demonstrated more varied and stronger results than females, who generally avoided looking at the face/head of stimuli. Moreover, Pazhoohi et al. (2019) found that males demonstrated longer dwell times on the chests of male images with higher shoulder to hip ratios than lower shoulder to hip ratios, while females demonstrated no differences in dwell times on the chests of any images. However, the focus of Pazhoohi et al.’s (2019) study was on ratings of attractiveness rather than attentional biases based on body image which may have influenced participants’ viewing patterns.

Unfortunately, not all studies identified in this review provided a clear consensus on the differences between males and females. In an eye-tracking study with German adults, Warschburger et al. (2015) found that women demonstrated longer gaze on attractive regions of interest than men, while men looked for longer at unattractive regions of interest. However, analyses of attentional bias (measured as a ratio of fixations on attractive/unattractive areas) did not detect significant differences between males and females. Overall, the data suggests different viewing patterns between males and females, and a higher percentage of fixations on unattractive regions for males (65.2%) than females (48.0%). Unfortunately, differences in attentional biases did not reach statistical significance (*p* = .230), making it difficult to draw concrete conclusions regarding the differences between males and females.

Moreover, some studies reviewed suggest there are no gender differences in attentional biases towards body-related stimuli. In an eye-tracking study with USA university students, Bernard et al. (2018) found that appearance-focused participants fixated on arms and stomachs of male images quicker and for longer; and faces for less time than personality-focused participants. However, fixation time effects were not moderated by gender, possibly because torso and arms are key indicators of physical attractiveness in males for both male and female audiences.

Overall, eye-tracking studies indicated that males demonstrated greater attentional biases towards muscular images (Cho & Lee, 2013; Jin et al., 2018; Porras-Garcia et al., 2019) compared to other body stimuli. While this may be similar to findings with females who view thinner images (e.g., Glauert et al., 2010; Prnjak et al., 2020), findings suggest that males and females demonstrate different viewing patterns (Hewig et al., 2008; Pazhoohi et al., 2019). These findings are indicative of not only different motivations for engaging with media ideal images, but also potentially different cognitive processes when engaged with them. However, the interpretation of eye-tracking data relies on supplementary assumptions from researchers, suggesting a potential gap between the original data and the conclusions drawn (Rahal & Fiedler, 2019). Therefore, caution should be taken when interpreting these results.

### Findings From Dot-Probe Studies

Dot-probe studies focussed primarily on height dissatisfaction amongst Chinese males (Cai et al., 2020; Chen, F. et al., 2017; Liu et al., 2014). While most literature examining attentional biases towards body-related stimuli in males focuses on weight/muscularity, other literature has focused on alternative areas of body image concern such as height. In a study examining stature dissatisfaction in male Chinese students, Liu et al. (2014) found that participants with high levels of stature dissatisfaction (HSD) demonstrated significantly slower reaction times in a dot-probe task when the probe followed a short-stature word compared to those with low levels of stature dissatisfaction (LSD). The HSD group also demonstrated greater recognition accuracy and higher recognition sensitivity for short stature words than the LSD group. Chen et al. (2017) built upon these findings in a study that combined a dot-probe task with eye-tracking. As with Liu et al. (2014), Chen et al. (2017) found that HSD participants demonstrated stronger attentional avoidance to short-related words than the LSD group, in addition to an initial avoidance bias from height-related words. Furthermore, compared to the LSD group, the HSD group had a shorter first fixation duration towards short-related words, and were more likely to disengage from height-related words. Findings from these studies seem to contradict findings from weight dissatisfied and eating disordered groups, who have demonstrated attention towards stimuli of concern rather than away (e.g., Cho & Lee, 2013; Joseph et al., 2016). However, similar patterns of avoidance have been demonstrated in studies of anxious participants (e.g., Garner, M., Mogg, & Bradley, 2006) in addition to studies with body dissatisfied males (e.g., Talbot et al., 2019).

Cai et al. (2020) contradict the previously discussed findings using a dot-probe task, electroencephalogram (EEG), and auditory height stimuli. Chinese adult males were exposed to an auditory cue (tall-related vs. short-related vs. neutral words) prior to each visual target of the dot-probe task. HSD participants demonstrated greater accuracy for visual targets preceded by tall-related words than LSD participants. Cai et al. (2020) suggest the differences in their findings and the findings of Liu et al. (2014) and Chen et al. (2017) may be down to the duration of exposure to the height stimuli. Within Liu et al. (2014) and Chen et al.’s (2017) studies exposure to stimuli was 1500 ms, while in Cai et al.’s (2020) study stimuli were presented for a much shorter length of 300 ms. It is proposed that longer exposure to threatening stimuli (i.e. short-related stimuli) leads to attentional avoidance, while the shorter exposure length in Cai et al.’s (2020) study was not adequate enough to produce this avoidance strategy.

Lane et al. (2019) also utilised a dot-probe paradigm, finding that there was no significant effect of dot-probe task (DPT) condition (neutral-cued vs. appearance-cued vs. time-delay) on attentional biases towards positive- or negative-appearance words in a sample of adult males. However, for participants who completed the appearance-cued DPT, attentional bias towards positive-appearance words was positively associated with a range of state variables, including perceived pressure from the media, internalisation of the leanness ideal and leanness dissatisfaction. From a social comparison perspective, it might be suggested that attending to positive-appearance words and viewing muscular images led participants to make upward comparisons to the attractive body stimuli which then led to greater body dissatisfaction and poorer mood (Festinger, 1954; Lane et al., 2019; Nikkelen et al., 2012). Alternatively, from a cognitive-behavioural perspective, it might be suggested that those experiencing body dissatisfaction experience maladaptive appearance-related schemas which result in cognitive biases (Lane et al., 2017, 2019; Markus et al., 1987; Rodgers & DuBois, 2016). Such biases include selective attention towards schema-relevant stimuli (i.e. stimuli that promote upward comparison and reinforce the negative appearance-related schema).

Overall, dot-probe studies highlighted that male body image concerns extend beyond muscularity concerns (Chen, F. et al., 2017; Liu et al., 2014). Interestingly, findings demonstrated attentional avoidance towards threatening stimuli (e.g. short-stature stimuli). These findings contradict those previously explored in this review, which suggested greater attention towards areas of concern. Future studies should explore this difference in more detail to determine if these patterns are due to differences in type of concern (height vs. muscularity) or another factor.

### Findings From Visual Search Studies

Surprisingly, only one study included within the current review utilised a visual search methodology. Using a compound visual search task, Talbot et al. (2019) demonstrated a positive correlation between obese-incongruent trial reaction times and body fat dissatisfaction, shape concern and weight concern. Those with higher levels of body dissatisfaction had quicker reaction times in trials where an obese image was paired with a distractor cue, suggesting these participants had developed strategies to avoid/ignore the obese images. Such cognitive avoidance supports a cognitive model whereby an individual will ignore/disregard information that does not fit with their schema of an ideal male body (Markus et al., 1987; Talbot et al., 2019). This avoidance is also analogous to the findings of dot-probe studies where participants demonstrated attentional avoidance of short-stature stimuli (Chen, F. et al., 2017; Liu et al., 2014).

### Findings Using Other Methodologies (ARDPEI Task, Attention Shifting Task, Memory Bias Task)

Remaining studies included in this review used alternative methodologies: the Attentional Response to Distal versus Proximal Emotional Information (ARDPEI) task (Dondzilo et al., 2021b), an attention shifting task (Liu et al., 2014), and a memory bias task (Unterhalter et al., 2007). Dondzilo et al. (2021b) used an ARDPEI task to assess engagement with/disengagement from muscular/non-muscular body images in a sample of Australian undergraduates. Results support a serial mediation model, whereby the relationship between increased attentional engagement and higher levels of body dissatisfaction is mediated by upward social comparisons prompted by engagement with muscular bodies (both peer and media images). These social comparisons were also associated with rumination that the individual does not meet the ideal appearance standards and higher levels of body dissatisfaction. This serial mediation model supports cognitive models of attentional biases towards body-stimuli, in addition to further highlighting the link between these biases body dissatisfaction (Dondzilo et al., 2021b).

In their second study, Liu et al. (2014) used an attention shifting task whereby participants were required to shift their attention from a centrally located stimulus (short vs. tall vs. neutral household words) to identify a peripherally located target. Following this task, participants were required to complete a word recognition task. Findings demonstrate that participants in the high stature dissatisfaction group were generally more accurate than the low stature dissatisfaction group when recognising short stature words. These findings are congruent with cognitive-behavioural theories that suggest information related to body image concerns is more likely to be encoded, and therefore recalled, than more generic information (Williamson et al., 2004).

This is further demonstrated in memory bias task findings from Unterhalter et al. (2007), whereby male undergraduate students recalled significantly more muscle words while female undergraduate students recalled significantly more weight-related words. Specifically, male participants recalled significantly more positive muscle-related words, while female participants recalled a comparable amount of negative and positive weight-related words. Unterhalter et al. (2007) suggest this may reflect some “cognitive protection from negative self-processing” (p. 387).

## Discussion

The current study provides a critical synthesis of the findings of existing studies exploring attentional biases in adult males towards body-related stimuli. This study builds upon that of Talbot and Saleme (2022) by drawing together the literature related to attentional biases towards body-related stimuli, including other areas of body image concern such as height, within males and providing a comparison of the attentional biases present in males/females and healthy males/males with body image pathologies. The current study also provides a full systematic review which follows PRISMA guidelines (Page et al., 2021), where previous reviews have not. Overall, the results of the current study suggest a specific pattern of attentional biases towards body-related stimuli in males experiencing body image concern. Such information is useful for clinicians and other health practitioners in developing effective interventions to reduce body image concerns in males.

It is widely agreed that attentional biases play a key role in body dissatisfaction (e.g., Jiang & Vartanian, 2018; Lane et al., 2017; Rodgers & DuBois, 2016). However, findings from the current review suggest that attentional biases towards body-related stimuli specifically affect males with body dissatisfaction/body image concerns, rather than the general population (Jin et al., 2018; Lane et al., 2019; Nikkelen et al., 2012; Porras-Garcia et al., 2020a). Predominantly, males with higher levels of muscle dissatisfaction demonstrated greater attention towards muscular images and muscle-related areas (Jin et al., 2018; Porras-Garcia et al., 2020b). Males with high drive-for-muscularity also demonstrated greater attentional biases towards self-reported attractive areas of their own body, while those with high drive-for-thinness demonstrated longer gaze towards self-reported unattractive areas of their own body (Cordes et al., 2016). In comparison, males at low risk of muscle dysmorphia/low muscle dissatisfaction demonstrated whole-body scanning patterns, rather than focussing on muscle-related areas specifically (Jin et al., 2018; Porras-Garcia et al., 2020b). However, all males demonstrated higher levels of attention towards attractive areas of muscular images, particularly the chest and abdomen (Cordes et al., 2016; Porras-Garcia et al., 2020b). These areas of the body relate to other dimensions of body image (dis)satisfaction, therefore it is important that future studies explore a range of dimensions beyond muscularity when exploring attentional biases towards body-related stimuli in males (e.g. body fat, slimness).

While most literature exploring attentional biases towards body-related stimuli in males has focussed on muscularity, some findings presented in this review surrounded alternative areas of body image concern (e.g., Cai et al., 2020; Chen, F. et al., 2017; Liu et al., 2014). For example, while body/muscle-dissatisfied males demonstrate stronger attentional biases towards muscular images, Talbot et al. (2019) found that body dissatisfied males demonstrated cognitive avoidance of obese images. These findings are indicative of a cognitive model, whereby an individual will avoid information that does not fit their schema of an ideal male body (Markus et al., 1987). Similar patterns of avoidance have been demonstrated in studies of anxious participants (Garner, M. et al., 2006) and in height-dissatisfied males, who demonstrate early processing of short-related words (Cai et al., 2020), but demonstrate attentional avoidance to threatening stimuli (i.e. short-related stimuli) when exposure duration is longer (Chen, F. et al., 2017; Liu et al., 2014).

The current review also explored the differences in attentional biases between healthy males with body image dissatisfaction and alternative groups such as those with body image pathologies or female samples. Findings suggest that participants with an MD diagnosis demonstrate significantly longer dwell times on subjectively attractive areas of hyper-muscular images (Waldorf et al., 2019). Research surrounding attentional biases with a male sample with body image pathologies is limited, but the general trends identified suggest similar, but stronger attentional biases to healthy controls with body image concerns. This may prove useful in the development of future interventions as similar interventions may work for both groups (healthy and body image pathologies), although more intensive interventions may be required to combat the stronger biases in those with diagnosed pathologies.

Most evidence (n = 5) included within the current review points towards gender differences in attentional biases when viewing body-related stimuli. Males demonstrated a preference for muscle-related stimuli while females showed a preference for thin/weight-related stimuli (Cho & Lee, 2013; Lane et al., 2019; Porras-Garcia et al., 2019; Unterhalter et al., 2007) Furthermore, males generally demonstrate more varied viewing patterns than females (Hewig et al., 2008). These findings highlight the importance of utilising male samples within this field of research. It is clear that males not only experience body image in a unique way but demonstrate different attentional biases towards body-related stimuli. Future research should look towards developing and utilising measures specifically for male samples that addresses their distinctive body image concerns.

The final aim of the current review was to identify the methodologies utilised in this field of research within a male sample. Most studies (n = 17) included in the current review have utilised eye-tracking methodologies (e.g., Cho & Lee, 2013; Cordes et al., 2016, 2017, Porras-Garcia et al., 2019, 2020b) or RT measures (e.g., Joseph et al., 2016; Lane et al., 2019; Liu et al., 2014; Talbot et al., 2019). Consequently, several authors have recommended combining RT and eye-tracking measures (Jiang & Vartanian, 2018; Uusberg et al., 2018). This allows researchers to explore the time-course of attention, obtain both cognitive and overt visual cues of attentional biases, and to further our understanding of the underlying attentional mechanisms of these biases.

### Strengths and Limitations

The current review has some limitations. Firstly, due to varied methodologies and designs utilised, it was not possible to conduct a full meta-analysis of the articles reviewed. This would be useful going forward to determine the overall effect of attentional bias studies with a male sample. Furthermore, as the current research project forms part of the first author’s doctoral thesis, literature searches and initial reviews were conducted by one researcher. However, the research team acted as additional reviewers of the risk of bias analyses to ensure no bias was introduced. The broad scope of this review also means many studies were included, so it is unlikely any biases have been introduced by the PI.

It is also important to highlight the limitations of examining attentional bias as a whole. Firstly, reaction time measures of attentional bias such as dot-probe and visual search tasks rely on the response latencies of participants to make judgements about attentional process (Jiang & Vartanian, 2018). Furthermore, it can be difficult to distinguish which attentional processes are being displayed within the attentional bias task results (Gao et al., 2011; Jiang & Vartanian, 2018). Finally, eye-tracking methodologies have been criticised in previous literature for not taking into account covert attentional processes (Blechert et al., 2010; Jiang & Vartanian, 2018).

Despite these limitations, the present study is the first systematic review to focus specifically on studies conducted with male participants. Previous reviews (e.g., Jiang & Vartanian, 2018; Rodgers & DuBois, 2016) have utilised studies mainly consisting of female only samples or have not followed PRISMA guidelines (Page et al., 2021; Talbot & Saleme, 2022). By focussing on male samples, the current systematic review can provide preliminary evidence of specific attentional biases towards body-related stimuli in body-dissatisfied males. These findings provide a robust foundation so evidence-based interventions can be targeted to this population.

There are some key considerations for future research. Firstly, it is important that appropriate scales and measurement tools for male participants are utilised. Moreover, future research should introduce additional factors such as participants’ reasons for engaging in social comparison (e.g., self-evaluation vs. self-improvement) and exercise (e.g., to improve appearance, athletic ability, health), as these factors have a direct impact on body (dis)satisfaction (Porras-Garcia et al., 2020b). Finally, it is recommended that future research combines measures of attentional biases, such as eye-tracking and reaction time measures, in order to evaluate both covert and overt mechanisms underlying these biases. By evaluating both mechanisms together, researchers can further unearth the underlying characteristics of these attentional biases (Jiang & Vartanian, 2018).

### Conclusions

The current study provides a critical synthesis of the findings of existing studies exploring attentional biases in adult males towards body-related stimuli. Overall, findings suggest that attentional biases towards body-related stimuli are specific to males experiencing body image concerns, and that these patterns are more prominent in those with body image pathologies. Furthermore, there appears to be a clear distinction between patterns of attentional biases in males and females. These findings are cross-sectional in nature; therefore, it is important that future research tries to establish the cause of attentional bias towards body-related stimuli in males, possibly through the use of attentional bias modification (ABM) procedures. Through exploring this potential causal relationship, effective interventions for targeting these maladaptive biases can be developed to improve body image for males experiencing body image concerns.
